# Comparison of Larval Therapy and Vacuum-Assisted Closure Therapy after Revascularization in Peripheral Artery Disease Patients with Ischemic Wounds

**DOI:** 10.1155/2022/8148298

**Published:** 2022-03-29

**Authors:** Ugur Cangel, Serhat Sirekbasan, Erdal Polat

**Affiliations:** ^1^Department of Cardiovascular Surgery, Medical Faculty, Bahcesehir University, Istanbul, Turkey; ^2^Department of Medical Laboratory Techniques, Eldivan Vocational School of Health Services, Çankırı Karatekin University, Çankırı, Turkey; ^3^Department of Medical Microbiology, Cerrahpasa Medical Faculty, Istanbul University-Cerrahpasa, Istanbul, Turkey

## Abstract

**Objective:**

Even for very successful peripheral revascularization therapy, treatment is not complete until the ulcerative, gangrenous, and infected wound is closed. This study was performed and compared the outcomes of vacuum-assisted closure (VAC) and maggot debridement therapy (MDT) following peripheral revascularization to accelerate the wound healing process.

**Methods:**

We did a prospective randomized clinical trial between January 1, 2014, and June 21, 2019. This study included 72 patients (63 males and nine females). Balloon angioplasty was performed in 21 patients (29.2%), peripheral bypass in 39 (54.2%), and both balloon angioplasty and revascularization (hybrid) surgery in 12 (16.7%). Thirty-three patients (45.8%) received 15 VAC therapy sessions for a month. Therapy progress was monitored at 48 h intervals, and wound debridement was performed. Thirty-nine patients (54.2%) received an average of six larval therapy sessions for a month. Groups were compared with the *X*^2^ test, and a statistically significant difference was found (*P* < 0.001).

**Results:**

In the VAC therapy group (*n* = 33), 14 patients (42.4%) had their feet amputated, 5 (15.1%) had a toe amputated, and 4 (12.1%) had all of their toes amputated. A skin graft was performed on four patients (12.1%) who developed granulation tissue. The wounds of six patients (18.2%) undergoing VAC therapy healed. In the larval therapy group (*n* = 39), the wounds healed in 36 patients (92.3%), and 3 (7.7%) had a toe amputated.

**Conclusion:**

Larval therapy was shown to be more effective than VAC therapy for the treatment of postrevascularization ischemic wounds. Thus, larval therapy can be used as an effective biological treatment method when major amputation is not required.

## 1. Introduction

Cardiovascular disease is one of the most significant complications in individuals with diabetes and is responsible for 50% of morbidity and mortality in adults. Vascular atherosclerosis is diagnosed much earlier than diabetes. Ulcerative and gangrenous lesions in patients with atherosclerosis peripheral arterial disease are resistant to treatment, particularly in the presence of an infection associated with uncontrolled diabetes. This condition often leads to amputation. Intensive antibiotic therapy for limb salvage in these individuals, who are generally elderly, suffer from multivascular atherosclerosis, and have infectious ischemic wounds, may cause these patients to die [1].

Even when very successful peripheral revascularization is performed on these patients, the therapy is not complete until the ulcerative, gangrenous, and infected wound is closed. Therefore, supportive therapies such as antibiotics, surgical debridement, hyperbaric oxygen (HBO), vacuum-assisted closure (VAC), hirudotherapy, and maggot debridement therapy (MDT) must be implemented to facilitate wound closure and accelerate the closure process after peripheral revascularization [2–4].

VAC therapy is frequently used as an alternative to traditional wound treatment. Most clinicians have reported decreased infection rates and thus shorter wound closure times with VAC therapy compared with traditional wound care methods [5–7].

Various studies that were performed with first- and second-stage larvae belonging to the *Lucilia sericata* fly have shown that MDT is an effective method for cleaning chronic wounds and granulation formation [8, 9]. Larvae, which do not harm healthy tissues, melt and remove necrotic tissues with the enzymes they secrete and disinfect the wound by eating, killing, and preventing the reproduction of microorganisms. Wound healing is faster with MDT than with classic therapy because MDT application forms a new, healthy tissue layer on the wound. Recently, MDT has been successfully used in the treatment of difficult-to-heal wounds on the foot caused by poor venous circulation [10, 11].

In the present study, we aimed to compare the effectiveness of VAC and MDT therapies. Thus, we applied either MDT or VAC to patients with peripheral artery disease and an ischemic ulcer on a lower extremity who underwent peripheral revascularization [12, 13].

## 2. Materials and Methods

### 2.1. Patients and Study Protocol

**Figure 1:**
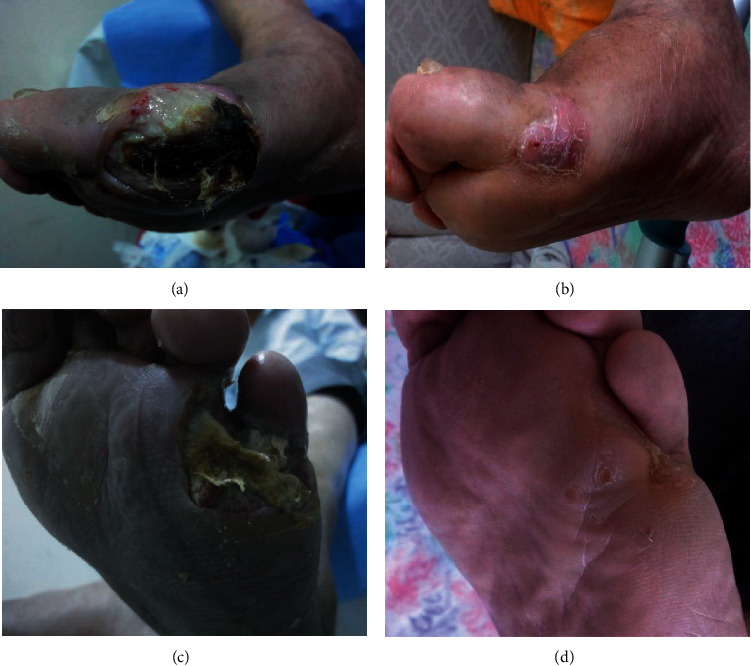
Two patients who underwent larvae treatment and whose wounds completely closed. (a) Patient 1-Before larva treatment. (b) Patient 1-After larvae treatment. (c) Patient 2-Before larva treatment. (d) Patient 2-After larvae treatment.

This study was performed in 72 patients who had peripheral artery disease with an ischemic ulcer on their lower extremities and underwent peripheral revascularization between January 2014 and June 2019. We conducted a prospective randomized clinical trial between January 1, 2014, and June 21, 2019. This study included 63 males (87.5%) and nine females (12.5%) with a mean age of 61 ± 10 years. Sixty-three patients (87.5%) had atherosclerosis, 57 patients (79.2%) had diabetes mellitus, 18 (25%) had chronic kidney failure, 11 (15.3%) had chronic obstructive pulmonary disease (COPD), 11 (15.3%) had coronary artery disease, and 9 (12.5%) had Buerger's disease. Lesions were located on the toe in 51 patients (70.8%), on the foot dorsum in nine (12.5%), between the heel and toes in six (8.3%), both on the heel and toe in three (4.2%), only on the heel in two (2.8%), and all over the foot in one (1.4%; Table 1; Figure 1).

### 2.2. Surgical Technique

Balloon angioplasty was performed in 21 patients (29.2%), a peripheral bypass was performed in 39 patients (54.2%), and both balloon angioplasty and revascularization (hybrid) surgery were performed in a single session in 12 patients (16.7%). Balloon angioplasty was performed under sedation and local anesthesia in the iliac artery in three patients, in the iliac + common femoral artery (CFA) in two, in the CFA + superficial femoral artery (SFA) in four, in the SFA in 19, and in the trifurcation arteries in five.

The femoro-popliteal artery bypass and popliteal-tibial bypass surgeries were performed under spinal anesthesia. During the procedure, 1 g of cefazolin sodium was administered intravenously to all patients for prophylaxis. The surgical site was disinfected with iodine and covered with a sterile drape, and 5000 units of heparin were given to the patients before the clamp. 6/0 polypropylene sutures were used for anastomoses, and end-to-side anastomosis was preferred. After completing the anastomoses, the cross-clamp was removed, the air within the graft was removed, and surgery was completed by closing the layers appropriately. The autogenous saphenous vein was used in all 21 patients in whom a distal anastomosis was created to the trifurcation distal, and a composite graft was performed for one patient. Of those 22 patients in whom a distal anastomosis was created to the popliteal artery, the saphenous vein was used in eight patients, and a polyester mesh vascular graft was used in 14 others whose saphenous vein was previously used or was structurally not suitable. The saphenous vein was used in three out of four patients who underwent femoro-tibialis posterior bypass, and one underwent bypass surgery with a composite graft made with polyester mesh and a saphenous vein graft. Iliofemoral bypass surgery was performed with an 8 mm polyester mesh graft in six patients. In 12 cases, distal anastomoses were created after endarterectomy (one in the PFA, three in the trifurcation area, two in the tibialis anterior, and two in the tibialis posterior).

### 2.3. Therapy Methods

Thirty-three patients received an average of 15 sessions of VAC therapy over one month, and if necessary, wound debridement was applied during the follow-ups, which were performed at 48-h intervals. VAC therapy was applied for 22 of every 24 h. The sponge of the device was replaced three times a week at intervals of 48–72 h and more frequently for infected wounds [5, 6, 14].

Thirty-nine patients (54.2%) received an average of six larval therapy sessions over one month. Larvae were placed in the wounds twice a week and removed after 48 h. If necessary, wound debridement was performed during the follow-ups conducted at our clinic [8, 9, 15].

### 2.4. Ethical Approval

The approval for the study was granted by the Ethics Committee of Istanbul University, Cerrahpasa Medical Faculty, with an ID number of 14620. A written informed consent was obtained from each patient. The study was conducted in accordance with the principles of the Declaration of Helsinki.

### 2.5. Statistical Analysis

The data were analyzed by SPSS 25.0 (SPSS Inc., United States), and the results were expressed as the mean ± standard deviation (SD). Chi-square tests were used to present statistical differences between the VAC and MDT groups. All *P* values under 0.05 were considered statistically significant.

## 3. Results

The health status of the patients was retrospectively examined on a computer and classified as I, II, III, and IV according to the Fontaine classification. None of the patients presented with Fontaine stage I and II. There were 38 patients in stage III (18 (47.4%) VAC group and 20 (52.6%) MDT group), and 34 patients in stage IV (13 (92%) VAC group and 1 (8%) MDT group). The Fontaine stage of the two groups was statistically similar (*P* > 0.05).

Some patients in the MDT group suffered from itching and pain. The MDT group was more frequently atherosclerotic (47% vs. 40%, *P*=ns), but the VAC group was more likely to have a history of smoking (34% vs. 29%, *P*=ns). Diabetes mellitus (82% for VAC vs. 77% for MDT) was common in both groups, but without significant differences between them. In addition, in terms of the patients' vasculopathy, we found no statistically significant difference between the two groups.

Almost all patients were at risk of amputation pretherapy. In the VAC therapy group, which included 33 patients, foot amputation was performed in 14 (42.4%), single toe amputation was performed in five (15.1%), and all toes were amputated in four patients (12.1%). Granulation tissue was formed within a month, and a skin graft was performed in four patients (12.1%) who underwent wound debridement with VAC therapy. Six (18.2%) of the patients who underwent only VAC therapy had their wounds completely closed. In the larval therapy group, which included 39 patients, 20 (51.3%) developed granulation tissue after 15 days, 14 (35.9%) developed granulation tissue after one month, and the other 2 (5.1%) developed granulation tissue later on. Three patients (7.7%) had a toe amputated due to circulatory failure. There was a statistically significant difference between the MDT and VAC groups in terms of toe amputation (*P* < 0.001).

## 4. Discussion

This study was performed on 72 patients who had peripheral artery disease with an ischemic ulcer on the lower extremities and underwent peripheral revascularization between January 2014 and June 2019. The major causes of peripheral artery disease were hyperglycemia, hypertension, smoking, genetic factors, and high levels of cholesterol. Hyperglycemia can cause serious damage if diabetes is not controlled. Organs and organ systems such as the heart, blood vessels, nervous system, kidneys, and eyes are affected over time. It causes 50% of the patients to die, as it increases heart disease and stroke risks [1]. In this study, 57 patients (79.2%) had diabetes mellitus and 18 (25%) had chronic kidney failure. Poor blood circulation, neuropathy, ulcers, and infections in the feet may lead to amputation. Therefore, the early diagnosis of diabetes, effective glycemic control, treatment of dyslipidemia, prevention of obesity, and dietary improvements, along with proficient diabetes education, will reduce vascular complications [16]. The most common vascular complications are wounds caused by ischemia at the extremities. Complications related to distal peripheral artery stenosis or occlusion increase with neuropathy. Burn wounds may occur in hyperglycemic patients as a result of footwear trauma or applying hot water bags to the feet because these patients suffer from neuropathic sensory loss. Lesions were located on the toe in 51 patients (70.6%), on the foot dorsum in nine (12.5%), between the heel and toes in six (8.3%), on both the heel and toe in three (4.2%), only on the heel in two (2.8%), and all over the foot in one (1.4%).

One of the five major risk factors of atherosclerosis is smoking. Atherosclerosis along with hypercholesteremia occurs as a result of trauma in the vascular walls caused by the ingredients in cigarettes. The fact that significantly more males (63, 87.5%) than females (9, 12.5%) were included in the present study may be related to the fact that smoking is more common in men than in women.

The most important therapy criterion in peripheral artery patients is the wound, and the wound also determines the treatment strategy. If there is no wound, then conservative treatment can continue to be applied in peripheral artery patients, and claudication distance can be increased with drug therapy. In the presence of a wound, interventional peripheral therapy becomes mandatory to prevent an amputation. In general, Fontaine stage I patients can recover after a simple peripheral intervention. Fontaine stage II and above patients require wound care. Patients are classified as Fontaine stage I, II, III, and IV. If subsystem issues progress to later stages and cardiac EF is equal to or less than 30%, then amputation must be considered rather than peripheral intervention. If there are subsystem issues in patients suffering from wounds on their toes, a toe amputation is first performed, the wound is left open, and medical treatment is administered. The interventional peripheral procedure is performed after the subsystem issues are corrected with medical treatment. Meanwhile, larval therapy can be used to prevent the progress of ischemia in the wound [17, 18]. Larvae melt, eat, and debride the necrotic tissues in a fast and effective way by using their proteolytic enzymes without harming the living tissue. Larvae, which do not like light, easily enter into places where a surgeon's scalpel cannot reach and remove dead tissues by melting them [15, 19–21]. Larvae disinfect the wound by applying bacteriostatic and bactericide effects on bacteria in the wound thanks to the hydrophobic peptide-like 3–10 kDa and hydrophilic 1 kDa antibacterial materials they produce by eating the microorganisms in the wound [15, 22]. Larvae also initiate tissue granulation and stimulate growth factors, thus enabling quick healing of the wound [8, 23]. Of the 39 patients who underwent larval therapy, the wounds fully closed in 36 patients (92.3%). The wounds did not heal in three (7.7%) patients because of circulation failure in the foot, which resulted in amputation of the toes.

In VAC therapy, the pain occurring during dressing changes generally decreases as the frequency of dressing changes decreases. As edema in the tissue resolves, the reproduction of bacteria slows down, and infection risk therefore decreases. VAC therapy stimulates the tissue, increases blood flow, and accelerates the wound healing process [5, 6, 24, 25]. VAC therapy has significantly better success compared with conventional wound care treatments [13]. Exudate absorption and granulation development in the wound help close the wound in indicated patients. In the VAC therapy group, which included 33 patients, 14 (42.4%) had their feet amputated, 5 (15.1%) had a single toe amputated, and 4 (12.1%) had all of their toes amputated. A skin graft was performed in four patients (12.1%) who underwent VAC therapy along with surgical debridement. The wounds of six patients (18.2%) were completely closed in the group that only underwent VAC therapy.

VAC therapy has some disadvantages [5, 6, 13]. For example, it does not replace debridement in a necrotic and infected wound. Conversely, larvae melt, eat, and debride the necrotic tissues in a fast and effective way by using their proteolytic enzymes without harming the living tissue. Larvae initiate tissue granulation and stimulate growth factors, while granulation tissue does not develop on the plates used for fixation in VAC therapy [8, 22]. VAC therapy is not used for peripheral artery disease-related wounds, while larval therapy can be used in all open wounds regardless of the underlying causes [9]. There may be pain and tickling in some patients undergoing larval therapy.

The pain completely disappeared after two to three sessions of larval therapy in patients who had chronic pain, persistent pain despite taking painkillers, Buerger's disease, venous insufficiency, and superficial wounds. VAC therapy was stopped if there was no change after two dressing changes or after a week, and the patient was referred to a cardiovascular surgeon if there was no positive change after two to three sessions of larval therapy. Larval therapy was continued after the circulation problem was eliminated. VAC therapy is an expensive method that should be used in a cost-effective manner along with conventional wound care treatment. Conversely, the cost of larval therapy is almost zero. Furthermore, larval therapy does not require hospitalization or nursing services. The patient can return to his/her daily life after larval therapy. Because no air should be let into the area to form a good vacuum in VAC therapy, this may result in an anaerobic infection and even internal rotting of the foot [5, 6, 24]. Larvae, which do not like light, easily enter into areas where a surgeon's scalpel cannot reach and remove dead tissues by melting them [15, 19–21].

## 5. Conclusions

In conclusion, six of the 33 patients (18.2%) who underwent only VAC therapy had their wounds completely closed, whereas 36 of 39 patients (92.3%) who underwent MDT therapy had their wounds closed. The *X*^2^ test revealed a very significant statistical difference between the MDT group and the VAC therapy group (*P* < 0.001). Thirty-three (45.8%) of the 72 patients included in the present study preferred VAC therapy, whereas 39 (54.2%) preferred MDT. This was because the patients would remain in bed because of the vacuum machine in the case of VAC therapy.

As shown in the abovementioned comparisons, MDT therapy is a more successful and inexpensive method than VAC therapy, and it is a complementary treatment method that can be used in cases where major amputation is not required in patients with postrevascularization ischemic wounds.

## Figures and Tables

**Table 1:** Patient characteristics.

Age (years)	61 ± 10	
Sex	M (63)	F (9)
*Underlying diseases*		

Atherosclerosis	63	87.5%
Diabetes mellitus	57	79.2%
Chronic kidney failure	18	25.0%
Coronary artery disease	11	15.3%
Chronic obstructive pulmonary disease	11	15.3%
Buerger	9	12.5%
Obesity	7	9.7%
Cerebral palsy	2	2.8%
Smoker	63	87.5%
Nonsmoker	9	12.5%

*Wound site*		

Toe	51	70.8%
Foot dorsum	9	12.5%
Between the toes and heel	6	8.3%
Heel and toe	3	4.2%
Heel	2	2.8%
All over the foot	1	1.4%
Ankle-brachial index (mean)	0.45 ± 0.9	
Ankle pressure (mmHg) (mean)	65	

## Data Availability

The data used to support the findings of this study are available from the corresponding author upon request.
